# Synthesis of high-specific-surface-area analcime from coal fly ash for hydrogen storage without external silica–alumina sources

**DOI:** 10.1039/d6ra05131f

**Published:** 2026-09-24

**Authors:** Dilinuer Aili, Aikelaimu Aihemaiti, Peng Yan, Abdukader Abdukayum

**Affiliations:** a Xinjiang Key Laboratory of Novel Functional Materials Chemistry, College of Chemistry and Environmental Sciences, Kashi University Kashi 844000 PR China abdukadera@sina.com

## Abstract

To address the valorization of coal fly ash and the limited hydrogen storage performance of zeolitic materials, a modified hydrothermal synthesis process is presented for producing analcime (ANA) with greatly-reduced crystalline impurities using pretreated coal fly ash without the addition of external silica–alumina sources. The ANA sample crystallized for 24 h at 375 K delivers a remarkably high specific surface area of 197.8 m^2^ g^−1^ together with a hydrogen-storage capacity of 0.57 mmol g^−1^. A positive correlation between specific surface area and hydrogen-adsorption capacity is demonstrated. This work provides a low-cost and efficient approach to the valorization of coal fly ash and further explores the hydrogen-storage potential of waste-derived analcime zeolite.

## Abstract 2

Zeolite analcime with high specific surface-area was directly synthesized from coal fly ash without extra silica–alumina sources, and its hydrogen-adsorption behaviour was investigated.

## Graphical abstract

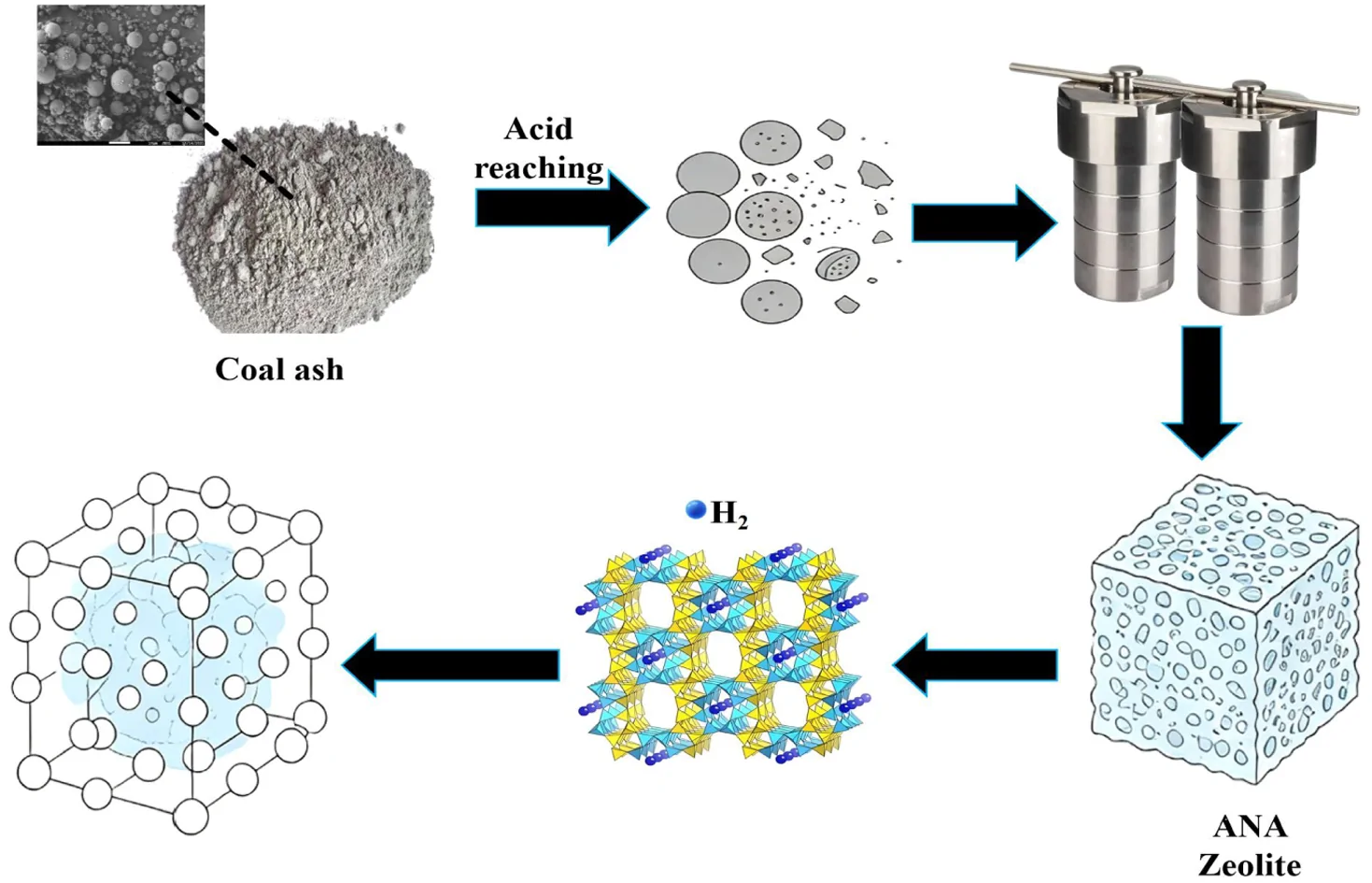

## 1. Introduction

Coal remains the dominant fuel for industrial applications in China, and its combustion generates substantial fly ash. With the rapid development of China's national economy, coal ash production has increased year by year. Although approximately 80% of coal ash is recycled, its total output continues to grow annually.^1^ Excessive stockpiled coal ash occupies vast land resources and causes environmental pollution, posing potential hazards to human health.^2^ Therefore, the efficient and high-value utilization of coal ash has become a critical research issue. Driven by this practical demand, numerous industries have focused on the resource utilization of coal ash,^3^ particularly in the preparation of molecular sieves,^4^ cement and concrete fillers,^5^ active enhancers for improving soil quality,^6^ and adsorbent materials for wastewater and exhaust gas treatment.^7^ Coal ash is primarily composed of amorphous aluminosilicates and crystalline phases including mullite, quartz, hematite and magnetite. Its spherical morphology, porous structure, low density and fine particle size facilitate the fabrication of low-silica zeolites.^8^ Synthesis of large quantities of porous zeolite material using coal ash has attracted extensive attention as a promising new technology to increase the utilization rate of solid waste.^9^

In recent years, zeolites have emerged as promising adsorbent materials for hydrogen storage.^10^ The superior advantages of zeolites as hydrogen storage carriers lie in their excellent thermal and chemical stability. Previous studies have investigated hydrogen adsorption behaviours in zeolite cages at room temperature, high temperature and high pressure conditions.^11,12^ Prabhakaran^13^*et al.* synthesized metal–organic frameworks (MOFs) and improved their hydrogen adsorption performance *via* lithium doping. Compared with zeolites, MOFs suffer from complicated synthesis procedures, low thermal stability and high preparation costs. Accordingly, zeolite materials have attracted extensive attention as competitive hydrogen storage adsorbents due to their high packing density and tunable chemical composition. Analcime (ANA), a low Si/Al ratio sodium aluminosilicate with a unit cell formula of Na_16_[AlO_2_]_16_[SiO_2_]_32_·16H_2_O and a Si/Al ratio ranging from 1.8 to 2.8,^14^ possesses unique internal structures and crystal chemical properties as a vital mineral and chemical raw material. ANA also exhibits excellent hydrothermal stability, adsorption capacity, molecular selectivity and ion exchange performance.^15^ Benefiting from these outstanding properties, ANA has been widely applied in soil improvement,^16^ heavy metal ion adsorption,^17^ wastewater treatment^18^ and catalytic reactions.^19^ However, there are limited studies focusing on the synthesis of ANA for hydrogen storage applications. This study innovatively synthesizes high-performance ANA for hydrogen storage using coal ash as the sole silicon and aluminum source. This strategy not only expands the resource utilization approaches of coal ash and reduces its land occupation and environmental pollution, but also realizes the high-value utilization of fly ash by producing high-performance zeolite products.

Hydrothermal synthesis is the most common and mature method for preparing ANA from fly ash and has been widely investigated. This approach is recognized as efficient, economical and environmentally friendly for ANA fabrication.^20,21^ Nevertheless, conventional synthesis strategies generally require prolonged crystallization time (24–72 h) and extra external silica–alumina precursors or exogenous activators to obtain analcime products.^22–25^ Wen *et al.*^22^ synthesized ZSM-5 zeolite using coal ash, silica and sodium hydroxide *via* orthogonal experimental design, and explored the crystallization performance at 150 °C with different durations (24, 36, 48, 60 and 72 h). The optimal hydrothermal synthesis conditions were determined as a crystallization temperature of 180 °C and a crystallization time of 36 h. Yao *et al.*^23^ prepared zeolite materials through the mechanochemical reaction of coal ash with stainless steel balls and NaOH over 24 h. Liu *et al.*^24^ adopted red mud and fly ash composite materials as raw materials, supplemented with NaOH and water glass activators, and successfully synthesized high-crystallinity ANA *via* a two-step alkali dissolution and hydrothermal treatment process with a crystallization time of 24 h. Liu *et al.*^25^ prepared ANA from fly ash through a simultaneous extraction method within 20 h, whereas the obtained product exhibited a significantly reduced specific surface area. In general, these traditional modification and synthesis methods show limited effects on improving the specific surface area of zeolite products. For example, zeolite P synthesized *via* hydrochloric acid treatment for 24 h only achieves a specific surface area^26^ of 42.0 m^2^ g^−1^, and ANA prepared by sodium hydroxide activation for 10 h merely reaches 8.0 m^2^ g^−1^. These results indicate that neither extending crystallization duration nor introducing auxiliary raw materials can effectively enhance the pore structure and specific surface area of zeolite products.

In this work, mixed-pore ANA with greatly-reduced crystalline impurities and remarkably high specific surface area was successfully synthesized in a short time by optimizing hydrothermal parameters, using only pretreated coal ash without additional external silicon–alumina sources. This finding breaks the conventional consensus that prolonged crystallization time contributes to higher specific surface area. The ANA sample prepared for 6 h achieves a specific surface area of 59.28 m^2^ g^−1^, which is comparable to that of Na-P1 molecular sieve (63 m^2^ g^−1^) synthesized over 48 h in a previous study. Notably, the sample obtained after 24 h of reaction exhibits a specific surface area of 197.76 m^2^ g^−1^, which is 4.7 times higher than that of acid-modified zeolite P (42 m^2^ g^−1^) reported in the literature. This facile and efficient strategy provides a novel and low-cost route for fabricating high-performance zeolites toward hydrogen storage, with a maximum hydrogen storage capacity of 0.573 mmol g^−1^. Although many previous publications have reported analcime preparation from coal fly ash, most studies mainly focused on material synthesis and characterizations for heavy-metal-removal applications. Systematic evaluation of hydrogen-adsorption performance for fly-ash-derived analcime remains relatively rare. The present work achieves analcime with remarkably high specific surface area from pretreated fly ash without external silica–alumina sources, and further investigates its hydrogen-storage behaviour, providing complementary experimental data for waste-to-energy-storage material development.

## 2. Experimental materials and methods

Raw coal fly ash was collected from Xinjiang Huadian Hongyanchi No. 2 Power Generation Co., Ltd. Hydrochloric acid (38 wt%), tetrapropylammonium hydroxide (TPAOH), and sodium hydroxide were purchased from Shaanxi Xi'an Chemical Reagent Plant and Tianjin Hedong Hongyan Reagent Factory, respectively. All chemical reagents used in this work were of analytical grade and employed directly without further purification.

### 2.1 Pretreatment of coal fly ash

Coal fly ash was first acid-leached with 5 mol L^−1^ HCl solution at 80 °C for 6 h to remove impurity elements including calcium and iron. The treated solid sample was then filtered, washed with deionized water to neutrality, and fully dried. Subsequently, the acid-purified fly ash was uniformly mixed with sodium hydroxide and thermally activated at 700 °C for 8 h to prepare the activated silicon–aluminum precursor for subsequent synthesis.

### 2.2 Synthesis of ANA

**Fig. 1:**
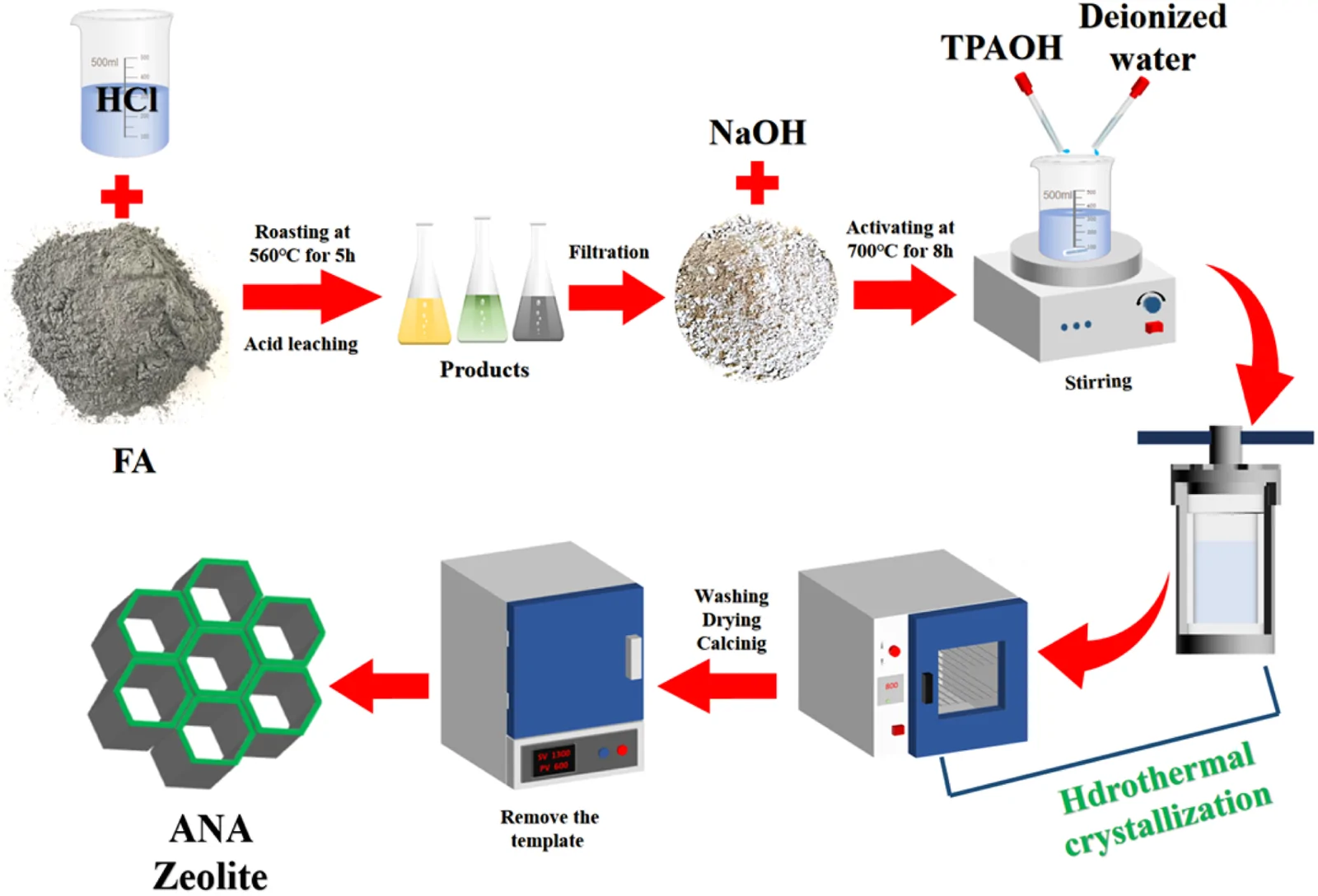
Schematic diagram of ANA preparation process.

The ANA zeolite was fabricated *via* a combined acid leaching-alkali fusion activation and hydrothermal method. Insoluble impurities in raw fly ash were removed by acid leaching, and the activated silicon–aluminum source was obtained through high-temperature alkali fusion activation. The hydrothermal reaction system was configured with a molar ratio of pretreated coal ash: TPAOH : H_2_O = 1 : 0.25 : 40, where TPAOH served as the structure-directing agent. The homogeneous mixture was then hydrothermally crystallized at 170 °C for 6–24 h. After the hydrothermal reaction, the resultant product was collected by filtration, dried, and calcined to obtain the final ANA zeolite sample. The detailed preparation route of ANA is illustrated in Fig. 1.

### 2.3 Hydrogen storage test

**Fig. 2:**
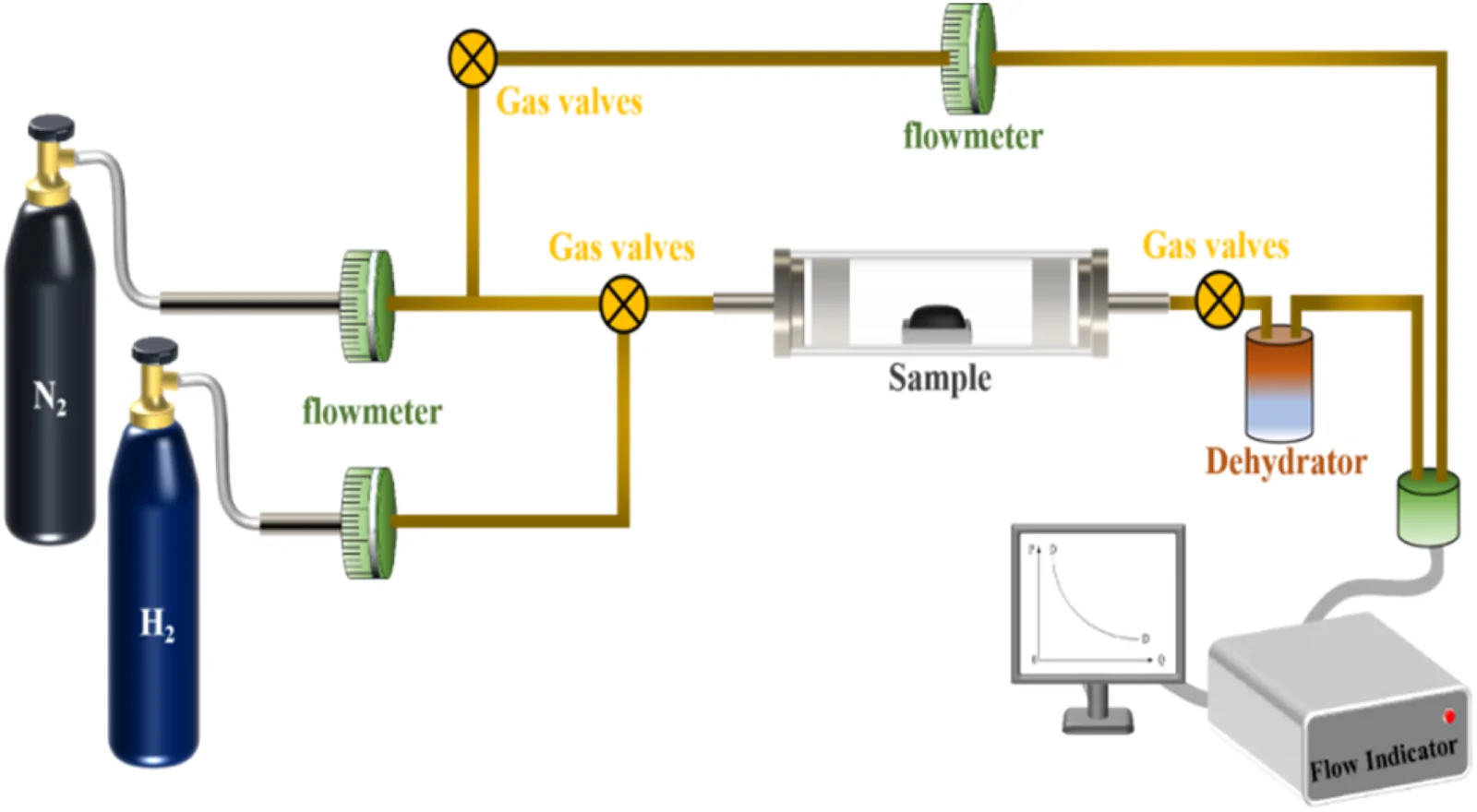
Hydrogen storage performance test device.

The hydrogen-storage performance of as-prepared ANA zeolite was evaluated by temperature-programmed hydrogen desorption (H_2_-TPD) using a TP-5076 automated chemisorption analyser (Fig. 2). The dried ANA sample was pelletised and sieved to obtain particle fractions of 0.25–0.425 mm. Approximately 0.1 g of sieved sample was loaded into a quartz tube and supported by quartz wool at the outlet side, with additional quartz wool placed at the inlet side. The sample was pre-treated under N_2_ atmosphere with a flow rate of 30 mL min^−1^, heated to 400 °C and held for 30 min, followed by cooling down to 25 °C. H_2_ adsorption was then carried out by introducing pure H_2_ adsorbate gas at 10 mL min^−1^ for 30 min. Afterwards, the system was purged with N_2_ for 30 min. Temperature-programmed desorption was subsequently performed from room temperature up to 900 °C at a heating rate of 10 °C min^−1^ to detect desorbed hydrogen. A blank experiment was performed under exactly the same instrumental parameters without loading any zeolite sample, and the blank signal was subtracted from the raw desorption curve. The desorption profiles were analysed using the matching TP5000 software, where the desorption peak area was integrated. The hydrogen-storage capacity was finally calculated by inputting the mass of the loaded zeolite sample. It should be noted that this capacity value is derived from H_2_-TPD and not from equilibrium adsorption–desorption isotherm measurements.

### 2.4 Characterization

X-ray fluorescence (XRF) was used to determine the elemental composition. X-ray diffraction (XRD) was used to analyse crystal phases. Scanning electron microscopy (SEM) was employed to observe sample morphology. N_2_ adsorption–desorption measurements were performed to obtain specific surface area and pore-size distribution *via* the BET method. Hydrogen-storage performance was evaluated by H_2_-TPD on a TP-5076 instrument.

The relative crystallinity of as-synthesized analcime was calculated from XRD patterns by peak-fitting integration using eqn (1):1
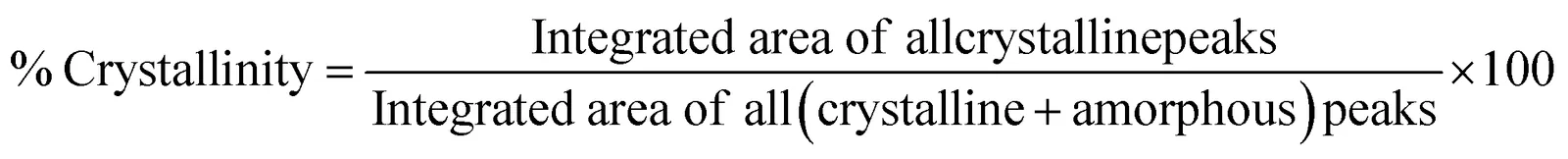


The calculation adopts the area-ratio method for zeolite crystallinity assessment.^26^ All XRD patterns were baseline-corrected before peak integration. The reported crystallinity value of 84.7% was calculated using this procedure.

## 3. Results and discussion

### 3.1 Effect of acid treatment concentration

The fly ash used in this study was collected from a thermal power plant in Changji, Xinjiang, China. It should be noted that coal fly ash is a highly heterogeneous material whose physical and chemical properties vary considerably depending on the source region, coal type and combustion conditions. Fly ash samples collected from different regions have been shown to exhibit distinct chemical compositions and acidic processing behaviours.^27,28^ Therefore, the formation of analcime and the resulting zeolite properties are strongly dependent on the specific characteristics of the raw fly ash employed. In particular, the SiO_2_/Al_2_O_3_ ratio of the raw material governs the framework composition available for analcime crystallisation, while the contents of impurities such as Ca and Fe determine the necessity and efficiency of the acid-leaching pretreatment. Accordingly, for fly ashes with substantially different compositions, the synthesis parameters (*e.g.*, acid-leaching conditions, Si/Al ratio adjustment and crystallisation temperature/time) would need to be re-optimised, and the present results are primarily applicable to the fly ash system investigated in this work.

X-ray fluorescence (XRF) analysis was performed to determine the elemental composition of raw coal ash and acid-treated coal ash, and the corresponding results are summarized in Table 1.

**Table 1:** Composition content of coal ash before and after acid treatment

Component content (wt%)	SiO_2_	Al_2_O_3_	Na_2_O	CaO	Fe_2_O_3_	MgO	SO_3_	TiO_2_	Si/Al
Before	36.80	20.75	5.70	24.51	6.33	4.07	3.79	0.73	1.50
After	35.76	10.95	4.23	2.34	4.79	1.76	0.65	0.11	2.77

The SiO_2_ and Al_2_O_3_ contents of raw coal ash account for 53.79% of the total components, which meets the silicon and aluminum source requirements for ANA zeolite synthesis. Nevertheless, the impurity contents (mainly CaO and Fe_2_O_3_) in raw coal ash reach 31.57%. These impurities reduce the effective Si/Al ratio and interfere with zeolite framework growth, thereby deteriorating ANA crystallinity and structure.^29^ Acid leaching effectively removes alkaline and amphoteric oxide impurities by converting them into soluble salts. Due to the chemical inertness of SiO_2_, only minor surface silicon loss occurs during acid treatment. Consequently, the Si/Al molar ratio increases from 1.50 (raw ash) to 2.77 after modification. This optimized ratio is favorable for the formation of ordered ANA Si–Al–O frameworks and significantly improves zeolite crystallinity.^30^

**Fig. 3:**
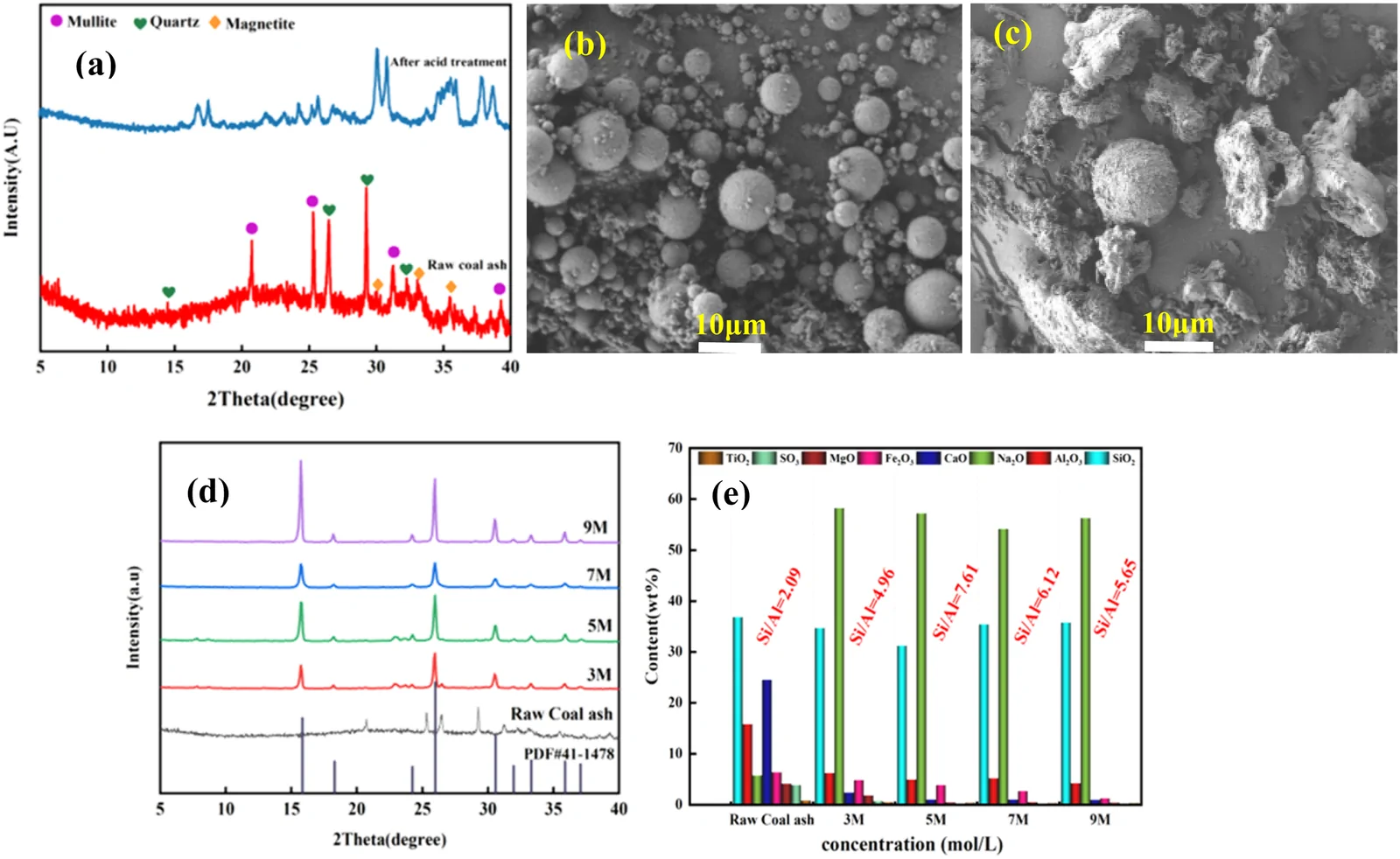
(a) XRD patterns of raw and acid-treated coal fly ash; SEM images of (b) raw coal fly ash and (c) acid-treated coal fly ash; (d) XRD patterns and (e) elemental composition of ANA samples synthesized at different acid pretreatment concentrations.

The structural variation of coal ash before and after hydrochloric acid treatment is demonstrated in Fig. 3. XRD patterns (Fig. 3a) reveal that raw coal ash is composed of an amorphous Si–Al matrix along with crystalline impurities, including mullite, quartz and magnetite, which is consistent with the multi-component impurity distribution obtained from XRF analysis in Table 1. After HCl acid leaching, all diffraction peaks corresponding to crystalline impurities disappeared. Combined with quantitative composition data, the contents of CaO and Fe_2_O_3_ decreased by 90.4% and 24.3%, respectively, and crystalline mullite phases were effectively removed. The acid-modified coal ash primarily presents an amorphous Si–Al precursor structure, which eliminates the phase competition effect during zeolite crystallization.

It is worth noting that the HCl washing step during sample preparation can effectively decompose and remove carbonate and bicarbonate species from the raw fly ash, since these components react with H^+^ to release CO_2_ under acidic conditions. This is supported by the significant decrease in CaO content from 24.51 wt% to 2.34 wt% after acid treatment (Table 1), indicating the efficient dissolution of calcium-bearing phases, including possible calcite (CaCO_3_). Consistently, no characteristic diffraction peaks assignable to carbonate minerals are observed in the XRD patterns of either the acid-treated precursor or the final analcime products. However, in the absence of FT-IR characterisation, trace amounts of amorphous or surface-bound carbonate/bicarbonate residues cannot be completely excluded, since such species may be present below the detection limit of XRD. Further FT-IR measurements are therefore recommended in future work to fully verify the absence of residual carbonate-related species.

SEM images (Fig. 3b and c) further verify the morphological reconstruction of coal ash after acid treatment. Untreated coal ash presents smooth spherical particles with a particle size of approximately 10 µm, and the particle surface is covered with fine impurity particles. This dense encapsulation structure restricts the dissolution and release of internal Si and Al species. After acid etching, the compact spherical structure is destroyed and transformed into loose aggregated fragments. The dissolution of Ca- and Fe-based oxides, together with the breakage of weak Si–O bonds by acid corrosion, contributes to the formation of a porous loose structure. This morphological change effectively increases the specific surface area and exposes abundant active reaction sites. Meanwhile, it accelerates the migration and rearrangement of Si and Al species during hydrothermal crystallization and reduces the nucleation energy barrier.

Several recent studies have demonstrated that trace amounts of iron, carbonate and bicarbonate in fly ash rapidly dissolve during acidic processing, which can lead to particle fragmentation and the formation of smaller particles.^31–33^ Such processes may partly mimic the HCl washing procedure employed in the present work. In our system, the contents of CaO and Fe_2_O_3_ decreased by 90.4% and 24.3%, respectively, after acid treatment (Table 1), confirming the effective removal of calcium- and iron-bearing phases. The dissolution of these soluble components generates voids and cracks within the ash particles, which partially contributes to the porous loose structure and the increased specific surface area observed by SEM (Fig. 3b and c). However, it should be emphasised that the porous loose structure is not solely a consequence of acid dissolution. The hydrothermal crystallisation of analcime itself generates a well-developed crystalline framework with intrinsic micro- and mesopores, as evidenced by the continuous increase of BET surface area with prolonged crystallisation time after the same acid pretreatment. Therefore, both acid-induced particle fragmentation and the formation of the analcime crystalline framework contribute to the final porous structure and high specific surface area, with the latter being the dominant contribution.

Overall, acid pretreatment achieves triple optimization of impurity removal, Si/Al ratio regulation and microstructure reconstruction. The elimination of impurities guarantees the structural regularity of the ANA zeolite framework; the loose porous morphology improves mass transfer efficiency; and the optimized Si/Al ratio matches the thermodynamic formation conditions of ANA. These synergistic effects lay a solid foundation for the subsequent synthesis of high-crystallinity ANA products.

### 3.2 Effect of acid treatment concentration

XRD patterns of samples obtained under different acid treatment concentrations are displayed in Fig. 3d. When the HCl concentration was 3 mol L^−1^, characteristic diffraction peaks of ANA appeared at 2*θ* = 15.8°, 18.3°, 24.3°, 25.9°, 30.5°, 32.0°, 33.2°, 35.8° and 37.1°. The peak positions and relative intensities are highly consistent with the standard PDF card (#41-1478, Fig. S1), confirming the successful synthesis of ANA zeolite. With the increase of acid concentration from 3 mol L^−1^ to 5 mol L^−1^, the intensity of ANA characteristic peaks gradually increased without detectable impurity peaks, and achieved optimal crystallization intensity at 5 mol L^−1^. Further increasing the acid concentration to 7 mol L^−1^ and 9 mol L^−1^ failed to continuously improve the peak intensity. This demonstrates that moderate acid treatment at 5 mol L^−1^ can effectively remove CaO and Fe_2_O_3_ impurities in coal ash, purify the Si–Al precursor, and remarkably promote the growth and crystallization of ANA crystals.

The component evolution of samples under gradient acid concentrations (Fig. 3e) shows that the Si/Al ratio increases significantly and reaches a maximum value of 2.77 at 5 mol L^−1^ HCl. However, further increasing the acid concentration to 7 mol L^−1^ and 9 mol L^−1^ leads to a gradual decline in the Si/Al ratio. Excessively high-concentration hydrochloric acid induces strong corrosive reactions, which destroy the stable SiO_2_ structure in coal ash and cause severe silicon loss. The relative enrichment of residual Al species ultimately reduces the Si/Al ratio.

Combined with crystallinity and component variation analysis, low-concentration HCl (3–5 mol L^−1^) mainly acts as a selective impurity remover, which eliminates Ca- and Fe-based crystalline interferences while retaining sufficient Si sources to optimize the Si/Al ratio for ANA synthesis. In contrast, excessive acid concentration (≥7 mol L^−1^) causes over-etching of the silicon phase, breaks the optimal elemental proportion of the Si–Al precursor, and is detrimental to ANA crystal growth. Accordingly, 5 mol L^−1^ is determined as the optimal acid treatment concentration in this work. This condition achieves a high Si/Al ratio and excellent ANA crystallinity without impurity interference, providing high-quality precursors for the subsequent hydrogen storage performance test of ANA zeolite.

### 3.3 Effect of crystallization time

**Fig. 4:**
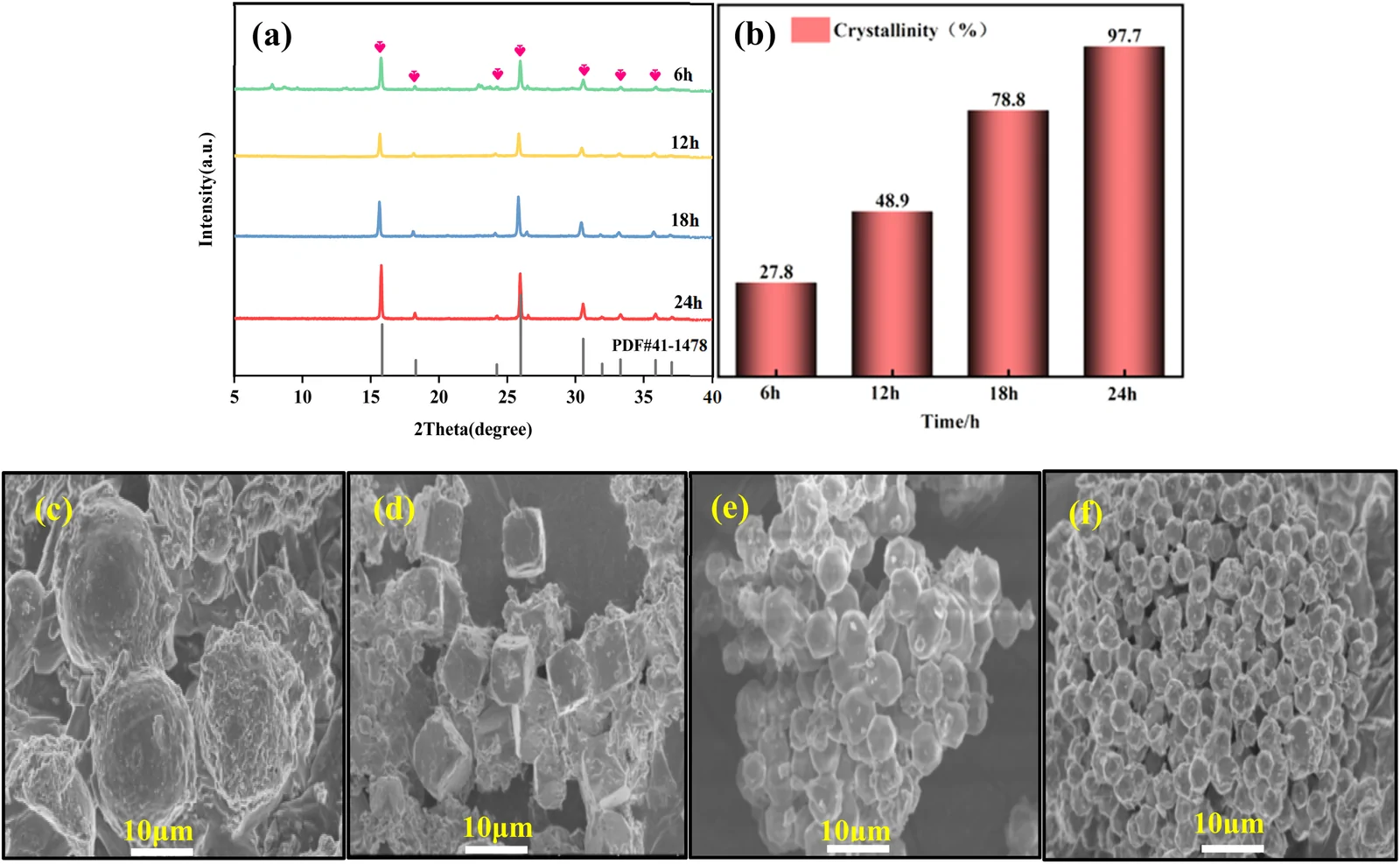
Characterization of ANA zeolites synthesized at different crystallization times. (a) XRD patterns of ANA samples obtained with varying crystallization durations; (b) corresponding crystallinity variation; (c–f) SEM images of ANA products synthesized for 6 h, 10 h, 14 h and 18 h, respectively (scale bar = 10 µm).

XRD patterns and corresponding crystallinity variation of ANA zeolites synthesized at different crystallization durations are displayed in Fig. 4. Weak characteristic diffraction peaks of ANA initially appear at 6 h (Fig. 4a), indicating that the reaction stays at the crystallization induction stage with insufficient crystal growth and low crystallinity. When the crystallization time extends to 12 h, the diffraction peak intensity rises significantly, and the corresponding crystallinity is 1.75 times higher than that of the 6 h sample. Further prolonging the reaction time to 18 h yields stronger and well-defined ANA characteristic peaks without detectable impurity phases. The crystallinity reaches nearly 85% at 18 h (Fig. 4b), confirming that appropriate extension of crystallization time effectively promotes ANA crystal growth. This trend is consistent with a previous study, which reported that prolonged hydrothermal duration benefits ANA crystallinity improvement within a reasonable reaction range. No obvious variation in diffraction intensity and crystallinity is observed when the crystallization time is further extended to 24 h, demonstrating that the crystallization reaction is basically completed at 18 h. Notably, high-crystallinity ANA can be successfully fabricated within 18 h using pure pretreated coal ash as the sole Si and Al source. This optimized strategy avoids the long-term crystallization duration (up to 72 h) and extra Si/Al precursor supplementation required in conventional synthetic routes.^34^

SEM images (Fig. 4c–f) further reveal the morphological evolution of ANA crystals at different crystallization stages with a uniform particle size of approximately 10 µm. The sample obtained at 6 h mainly consists of amorphous precursors without complete crystal formation (Fig. 4c). As the reaction proceeds to 10 h, partial precursor agglomeration occurs, forming irregular hexahedral structures stacked by flaky crystals (Fig. 4d). With continuous hydrothermal reaction, the irregular cubic precursors gradually evolve into regular dihedral crystal frameworks. Well-defined ditetrahedral ANA single crystals with uniform particle sizes are formed at 18 h (Fig. 4e). No distinct morphological changes are observed for the 24 h sample, which presents compact and stable crystal particles (Fig. 4f). This morphological evolution is highly consistent with the XRD crystallinity variation, further verifying that 18 h is sufficient for the complete crystallization of high-quality ANA zeolite in this system.

**Fig. 5:**
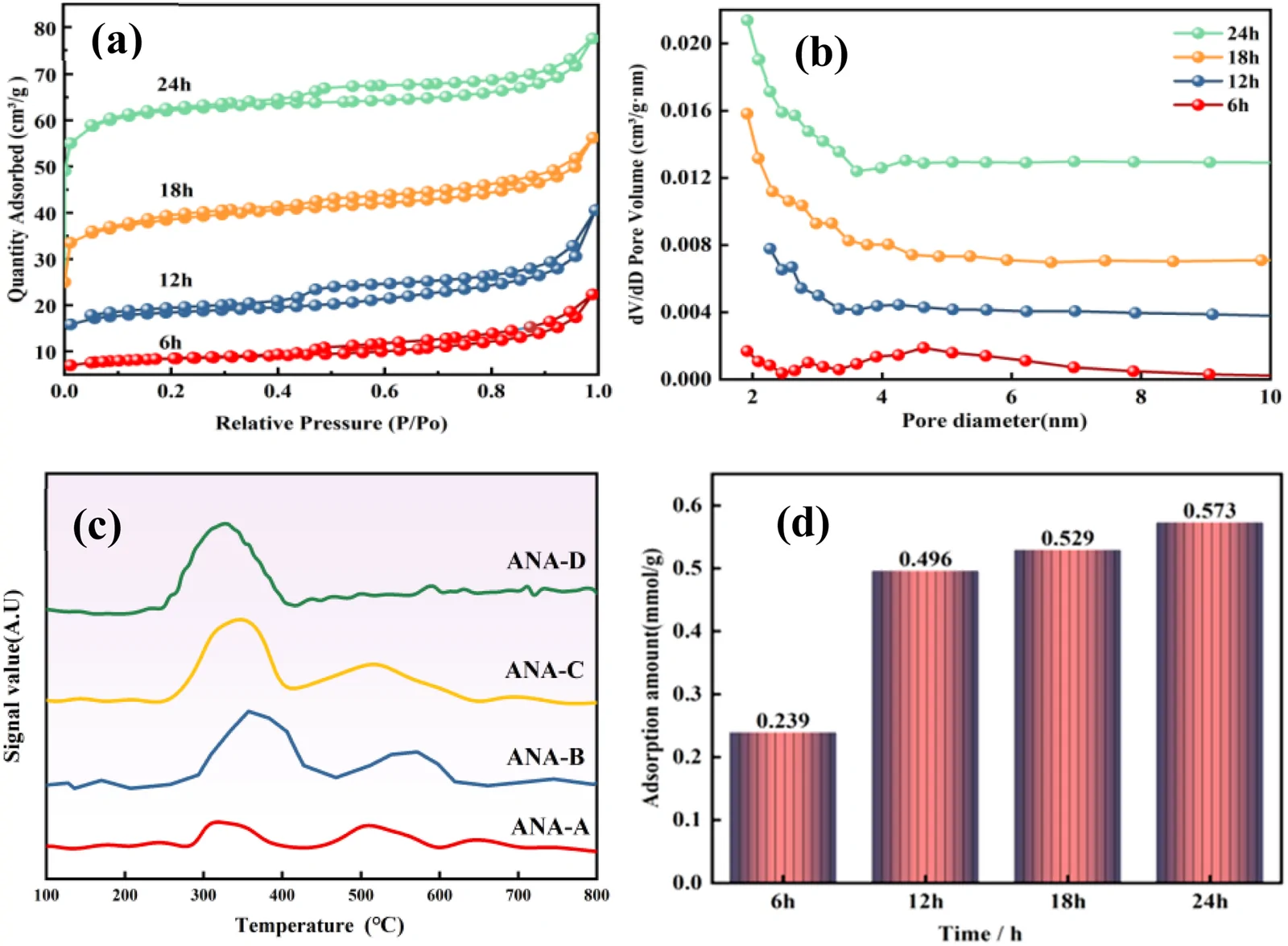
Pore structure and hydrogen storage performance of synthetic ANA zeolites. (a) N_2_ adsorption–desorption isotherm; (b) pore size distribution diagramand; (c) H_2_ -TPD curves of the as-prepared samples; (d) adsorption capacity.

The N_2_ adsorption–desorption isotherms and pore size distribution of synthetic ANA samples are displayed in Fig. 5, and the corresponding textual parameters are summarized in Table S1. As shown in Fig. 5a, the prepared ANA exhibits a mixed Type I and Type IV isotherm. A continuous rise in adsorption capacity is observed at a low relative pressure (*P*/*P* < 0.4), demonstrating the existence of micropores. A sharp increase in adsorption uptake at *P*/*P* > 0.8 and a distinct hysteresis loop in the range of 0.5–1.0 further confirm the formation of mesoporous structures. Corresponding pore size distribution curves in Fig. 5b further verify the hierarchical pore structure of synthetic ANA. The samples present a concentrated mesopore distribution centered at 2.43 nm, accompanied by abundant micropores, which confirms the coexistence of micro- and mesoporous structures. This unique hierarchical structure facilitates the diffusion of reactants and products and effectively inhibits carbon deposition during adsorption reactions.

As listed in Table S1, the specific surface area of ANA increases gradually with prolonged crystallization time, ranging from 59.28 m^2^ g^−1^ to 197.76 m^2^ g^−1^, with a typical mesopore size of 2.43 nm. This indicates that adjusting the crystallization duration can regulate the surface morphology, pore structure and porosity of ANA zeolite. A large specific surface area can transform the single intracrystalline diffusion into the coupled Knudsen diffusion and intracrystalline diffusion mode, significantly improving molecular diffusion efficiency and providing a structural foundation for excellent adsorption performance.^35^

Table 2 compares the textual properties and synthesis conditions of the as-prepared ANA series (ANA-A to ANA-D) with various reported zeolites, including zeolite P,^25^ NaA,^36^ Na-P1, ANA, SOD^21^ and other conventional zeolite materials.^37^ All ANA samples fabricated *via* the optimized acid treatment–hydrothermal method possess excellent specific surface areas. Notably, ANA-D achieves the maximum specific surface area of 197.76 m^2^ g^−1^, which is more than 3 times higher than that of reported zeolite P (42.00 m^2^ g^−1^) and Na-P1 (63.00 m^2^ g^−1^). Even the lowest-value sample ANA-A (59.28 m^2^ g^−1^) is comparable to or superior to most reference zeolites. The remarkably enlarged specific surface area provides abundant active adsorption sites for hydrogen molecules, which is critical for improving the hydrogen storage capacity.^38^ It should be noted that the relatively high BET specific surface area of as-prepared analcime originates from multiple co-existing factors. According to the XRD patterns, obvious secondary crystalline impurity phases are absent, so their contribution to surface area can be neglected.^39^ Small amounts of residual amorphous aluminosilicate cannot be fully excluded from XRD amorphous halo; nevertheless, the continuous rise of BET surface area with increasing crystallinity indicates that residual amorphous material is not the dominant source.^40^ As observed from SEM micrographs, particle aggregation creates inter-particle voids, which contribute partly to the measured mesoporous surface area.^41^ Moreover, acid-etching pretreatment generates intra-crystal structural defects, providing additional inner surface sites.^42^ Consequently, the high surface area is a combined result of acid-induced crystal defects and inter-particle pores, with only minor influence from residual amorphous components.

**Table 2:** Comparison of textual parameters of fly ash-derived ANA zeolites prepared in this work with reported zeolite materials

Sample	*S*_BET_ (m^2^ g^−1^)	Method	Hydrothermal reaction time (h)	Application	
ANA-A	59.28	Treated with HCl -hydrothermal method	6	H_2_ adsorption	This work
ANA-B	125.58	Treated with HCl -hydrothermal method	12	H_2_ adsorption	This work
ANA-C	167.53	Treated with HCl -hydrothermal method	18	H_2_ adsorption	This work
ANA-D	197.76	Treated with HCl -hydrothermal method	24	H_2_ adsorption	This work
Zeolite P	42.00	Treated with HCl -hydrothermal method	24 h	—	25
NaA	17.38	Alkali fusion- hydrothermal method	6 h	CO_2_ capture	36
Na-P1	63.00	NaOH solution -hydrothermal method	48 h	Cs^+^ and Sr^2+^ removal	26
ANA	8.00	NaOH solution -hydrothermal method	10 h	Cs^+^ and Sr^2+^ removal	26
SOD	17.00	NaOH solution -hydrothermal method	10–20 h	Cs^+^ and Sr^2+^ removal	21
Zeolite	13.23	NaOH solution -hydrothermal method	20 h	Adsorption capacity of Cu, Pb, Cr, Ni, d in wastewater	37

### 3.4 Hydrogen storage performance of ANA

The low-temperature α-peak (100–400 °C) is assigned to physisorption originating from van-der-Waals interactions between hydrogen molecules and the zeolite surface. For the high-temperature β-peak (450–600 °C), we tentatively assign it to chemisorption on ANA framework-wall sites. This assignment is not solely based on desorption temperature. According to previous zeolite hydrogen-storage reports, chemisorption linked to Si–OH–Al bridging hydroxyls and structural defects usually occurs above 450 °C.^43^ In our sample series, the peak area of the β-peak increases with rising crystallinity; well-formed analcime frameworks provide more Si–OH–Al hydroxyl groups and structural defect sites, which can act as potential chemisorption sites for hydrogen.^44^ A desorption temperature above 450 °C also suggests an interaction strength much stronger than ordinary van-der-Waals physisorption. It should be noted that direct *in situ* spectroscopic evidence (*e.g.*, *in situ* DRIFTS or Raman) for adsorbed hydrogen species is absent in this work. Further *in situ* characterisations are needed to achieve definitive identification of hydrogen-adsorbed species. Low-temperature physisorption and high-temperature chemisorption synergistically contribute to the total hydrogen-storage capacity of fly-ash-derived analcime.

Fig. 5d intuitively presents the variation in hydrogen storage capacity of ANA samples obtained at different crystallization durations. The hydrogen-storage capacity of the optimised fly-ash-derived analcime reaches 0.573 mmol g^−1^ (approximately 0.12 wt% H_2_). It should be noted that this value originates from semi-quantitative H_2_-TPD rather than static equilibrium adsorption measurement. For unmodified metal-free aluminosilicate zeolites, hydrogen uptake under moderate temperature-pressure conditions is generally low. The obtained capacity is comparable with many reported pure zeolite materials under similar test conditions, yet lower than metal-doped zeolites and specialised commercial hydrogen-adsorbents. The highlight of this material lies in the reuse of coal fly-ash waste for low-cost analcime preparation, rather than ultra-high hydrogen-storage capacity. Further performance improvement could be realised by metal-species modification, hierarchical-pore construction and tuning of skeletal hydroxyl-defect sites.

Combined with the pore structure parameters listed in Table S2, the increase in crystallization time effectively enlarges the specific surface area and pore volume of ANA, which correspondingly improves the hydrogen adsorption capacity. As the specific surface area increases from 59.28 m^2^ g^−1^ to 197.76 m^2^ g^−1^, the hydrogen storage capacity rises significantly from 0.239 mmol g^−1^ to 0.573 mmol g^−1^, achieving a nearly two-fold enhancement. This trend clearly demonstrates that the optimized hierarchical pore structure and enlarged surface area provide sufficient active sites for hydrogen adsorption, which are the key factors for improving the hydrogen-storage performance of coal-ash-derived ANA zeolites.

To verify that the observed hydrogen-storage performance originates from the analcime phase rather than from fly-ash-derived impurities, two control samples, raw fly ash and acid-treated fly ash without hydrothermal treatment, were additionally characterised by H_2_-TPD under identical conditions. Both control samples show much lower hydrogen-storage capacity than the crystallised ANA products. In particular, the raw fly ash delivers only a weak desorption signal together with a weak high-temperature β-peak (450–600 °C) whose intensity is far below that of the optimised ANA sample. After acid treatment, this high-temperature β-peak essentially disappears, and the acid-treated fly ash shows only a limited, mainly low-temperature desorption response. Most importantly, the characteristic high-temperature β-peak (450–600 °C), which is prominent in the well-crystallised ANA samples, is drastically weakened in the raw fly ash and nearly absent in the acid-treated fly ash.The slight increase in hydrogen uptake from raw fly ash to acid-treated fly ash can be attributed to the porous structure generated during acid leaching, but this contribution is minor compared with that of the crystalline ANA framework. These results confirm that the major hydrogen-adsorption capacity is derived from the synthesised analcime phase (Fig. S2 and Table S3).

## 4. Conclusion

This work focuses on the green and efficient route to fabricate analcime zeolite with greatly-reduced crystalline impurities and remarkably high specific surface area from pretreated raw coal fly ash, without introducing external silica–alumina sources. Beyond material preparation, its hydrogen-storage performance is preliminarily evaluated to explore the application prospect of such waste-derived hierarchical-porous ANA for hydrogen-storage. In this study, high-performance hydrogen-storage analcime (ANA) zeolite was successfully fabricated using coal fly ash as the sole silicon and aluminum source without additional chemical additives. An optimized 5 mol L^−1^ HCl pretreatment effectively removed calcium and iron impurities, regulated the elemental composition, and increased the Si/Al ratio to 2.77, which reconstructed the precursor microstructure and benefited subsequent hydrothermal crystallization. The optimized ANA-D sample crystallized for 24 h exhibited a superior specific surface area of 197.76 m^2^ g^−1^, which was 4.7 times higher than that of conventional acid-modified zeolites reported in previous studies. Moreover, the ANA-A sample prepared within only 6 h achieved a comparable surface area to zeolite products obtained *via* traditional 48 h synthetic routes, demonstrating the high efficiency of the proposed strategy. Owing to its hierarchical porous structure and large specific surface area, the optimal ANA sample delivered a favorable hydrogen storage capacity of 0.573 mmol g^−1^ at 375 K.

This study provides a facile, low-cost, and sustainable route for the high-value utilization of fly ash solid waste and offers a novel insight into the design and preparation of high-performance zeolite adsorbents for hydrogen storage. Future work will focus on process scale-up, practical condition evaluation, metal doping modification, and extended gas adsorption applications including CO_2_ and methane capture. It should be pointed out that the hydrogen-storage capacity reported herein originates from semi-quantitative H_2_-TPD measurements rather than equilibrium hydrogen adsorption–desorption isotherms. Further work is required to measure equilibrium isotherms and assess adsorption–desorption reversibility, cycling stability, hydrogen release fraction, retention time and adsorption–desorption kinetics for comprehensive evaluation of practical hydrogen-storage performance. *In situ* spectroscopic measurements are also desirable to clarify the nature of high-temperature hydrogen-adsorbed species. Moreover, material modification strategies including metal doping, hierarchical pore fabrication and defect-site regulation are promising approaches to further upgrade its hydrogen-adsorption performance.

## Author contributions

Dilinuer Aili: writing – original draft, methodology, data curation, investigation, and formal analysis. Aikelaimu Aihemaiti: writing – review and editing, and formal analysis. Peng Yan: data curation and validation. Abdukader Abdukayum: writing – review and editing, supervision, resources, methodology, and investigation.

## Conflicts of interest

The authors declare no competing interests.

## Supplementary Material

RA-OLF-D6RA05131F-s001

## Data Availability

The data supporting the findings of this study are available within the article and its supplementary information (SI). Supplementary information: XRD reference-card comparisons, full specific-surface-area results, and original experimental test data for the analcime samples. See DOI: https://doi.org/10.1039/d6ra05131f.
