# Supplementary material for: Synthesis of high-specific-surface-area analcime from coal fly ash for hydrogen storage without external silica–alumina sources
Source: RSC Adv. 2026 Sep 24;16(45):57302–11. doi: 10.1039/d6ra05131f (PMC13609867; doi:10.1039/d6ra05131f)
Supplement: Supplementary file 1 [file RA-016-D6RA05131F-s001.pdf]

Supplementary Information (SI) for RSC Advances.
This journal is © The Royal Society of Chemistry

Supplementary Information

Synthesis of high-specific-surface-area analcime from coal fly ash for hydrogen storage without external silica-alumina sources

*Dilinuer Aili, Aikelaimu Aihemaiti, Peng Yan, Abdukader Abdukayum**

Xinjiang Key Laboratory of Novel Functional Materials Chemistry, College of Chemistry and Environmental Sciences, Kashi University, Kashi 844000, PR China.

**Corresponding author: abdukadera@sina.com*

Contents

Figure S1. standard card PDF#41-1478 for ANA 3

Table S1. BET data of different samples 3

Table S2. Specific surface area data and hydrogen storage capacity of different samples......4

Figure S2. H₂-TPD profiles of raw fly ash, acid-treated fly ash and analcime samples crystallized for different durations 4

Table S3. H₂ uptake capacities of raw fly ash, acid-treated fly ash and analcime samples obtained with different crystallization times, determined by H₂-TPD 5

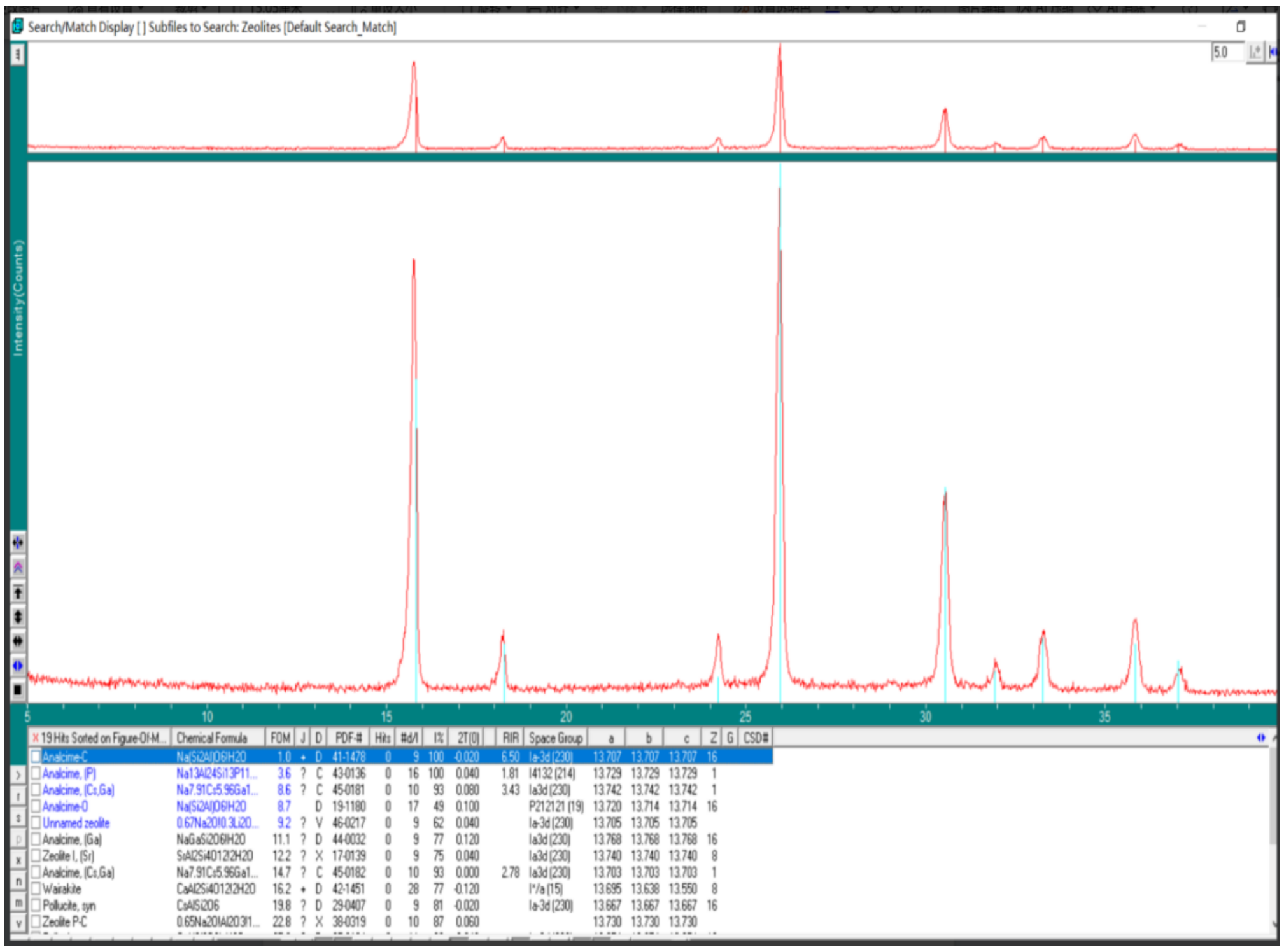

Figure S1. standard card PDF#41-1478 for ANA

Table S1. BET data of different samples

sample	SBET (m^2/g)	V_T (cm^3/g)	Vm (cm^3/g)	D (nm)
6h-ANA	59.2877	0.0212	0.0281	4.1438
12h-ANA	124.4814	0.0504	0.0586	2.7935
18h-ANA	167.5396	0.0700	0.0774	2.3601
24h-ANA	197.7656	0.0895	0.0952	2.4302

Table S2. Specific surface area data and hydrogen storage capacity of different samples

sample	S_{BET} (m^2/g)	V_T (cm^3/g)	Vm (cm^3/g)	D (nm)	Adsorption amount [mmol/g]
ANA-A	59.2877	0.0212	0.0281	4.1438	0.239
ANA-B	125.5814	0.0504	0.0586	2.7935	0.496
ANA-C	167.5396	0.0700	0.0774	2.3601	0.529
ANA-D	197.7656	0.0895	0.0952	2.4302	0.573

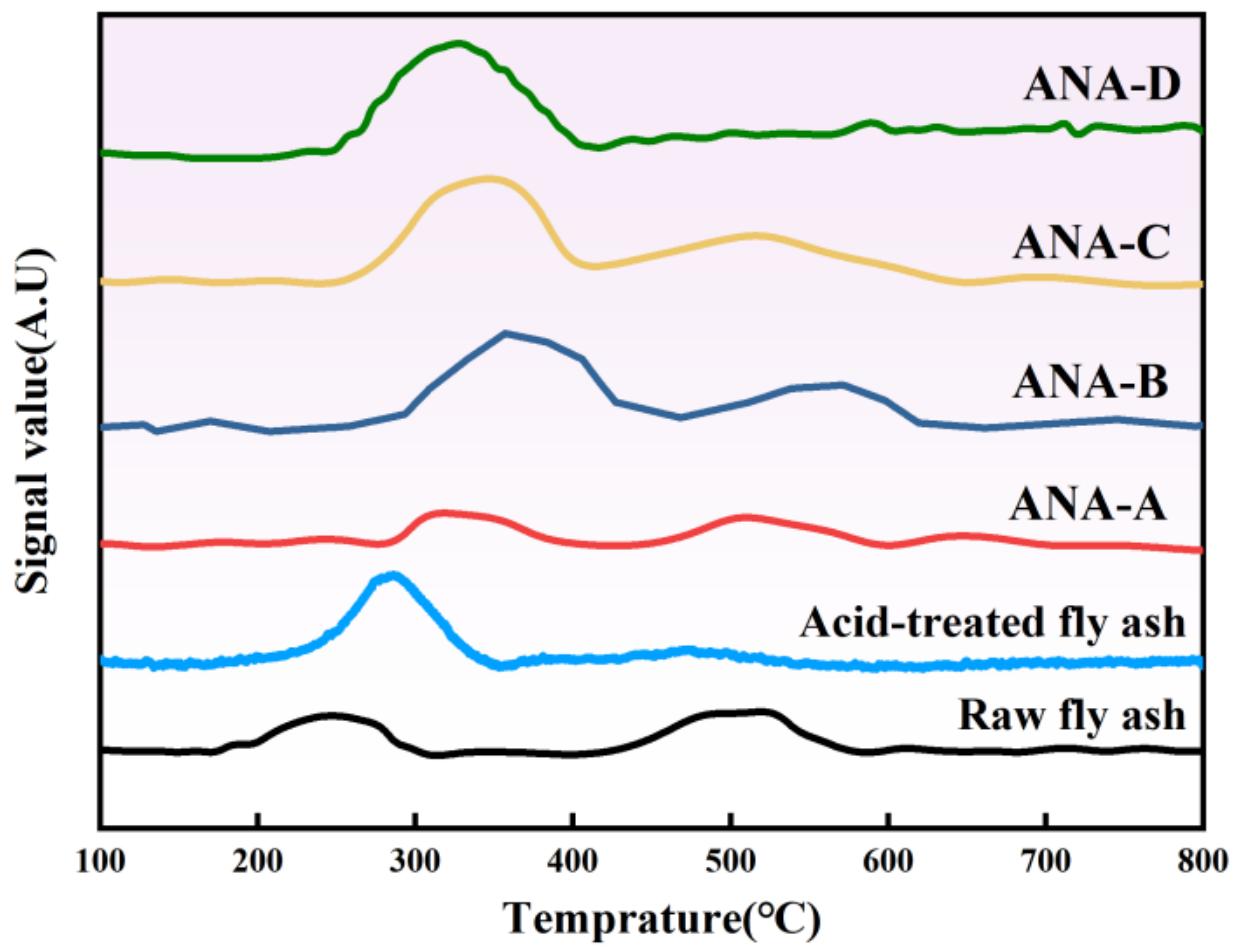

Figure S2. H_2-TPD profiles of raw fly ash, acid-treated fly ash and analcime samples crystallized for different durations

Table S3. H_2 uptake capacities of raw fly ash, acid-treated fly ash and analcime samples obtained with different crystallization times, determined by H_2-TPD

Sample	Crystallization time (h)	H_2 uptake ($mmol \cdot g^{-1}$)
Raw fly ash	-	0.100
Acid-treated fly ash	-	0.104
ANA-A	6	0.239
ANA-B	12	0.496
ANA-C	18	0.529
ANA-D	24	0.573
